# Imaging cellular forces with photonic crystals

**DOI:** 10.1038/s41467-023-43090-9

**Published:** 2023-11-14

**Authors:** Qiwei Li, Zaozao Chen, Ying Zhang, Shuang Ding, Haibo Ding, Luping Wang, Zhuoying Xie, Yifu Fu, Mengxiao Wei, Shengnan Liu, Jialun Chen, Xuan Wang, Zhongze Gu

**Affiliations:** 1 State Key Laboratory of Digital Medical Engineering, School of Biological Science and Medical Engineering, Southeast University, 210096 Nanjing, Jiangsu China; 2 Institute of Biomaterials and Medical Devices, Southeast University, 215163 Suzhou, Jiangsu China; 3 Faculty of Sports Science, Ningbo University, 315211 Ningbo, China

**Keywords:** Assay systems, Biosensors, Optical imaging, Bioinspired materials

## Abstract

Current techniques for visualizing and quantifying cellular forces have limitations in live cell imaging, throughput, and multi-scale analysis, which impede progress in cell force research and its practical applications. We developed a photonic crystal cellular force microscopy (PCCFM) to image vertical cell forces over a wide field of view (1.3 mm ⨯ 1.0 mm, a 10 ⨯ objective image) at high speed (about 20 frames per second) without references. The photonic crystal hydrogel substrate (PCS) converts micro-nano deformations into perceivable color changes, enabling in situ visualization and quantification of tiny vertical cell forces with high throughput. It enabled long-term, cross-scale monitoring from subcellular focal adhesions to tissue-level cell sheets and aggregates.

## Abstract (web summary)

Current techniques for visualizing cell generated forces suffer from throughput limitations. Here, Gu et al. introduced photonic crystal cellular force microscopy, inspired by chameleons, enabling visualization and quantification of vertically directed cell forces, well-suited for drug screening.

## Introduction

Cells exert, sense, and respond to their microenvironment through complex and numerous varieties of mechanisms. Cellular forces regulate physiological functions on multiple scales^1,2^, such as cellular proliferation and differentiation^3–5^, tissue or organ level development^6–8^, homeostasis maintenance^9–11^, immune recognition and killing^12–14^, etc. It is also related to various pathological changes, such as cardiomyopathy^15,16^, fibrosis^17–19^, and cancer^20,21^, making cellular force an indicator in drug screening^22^. Therefore, measuring cellular forces in situ, high-throughput, and cross-scale, low interference is of great importance for fundamental research and applications in drug development.

Cellular forces analysis started from semi-quantitative data, which were acquired by analyzing cell-generated wrinkles on elastomeric substrates^23^. Afterward, methods based on high-quality displacement field measurement to calculate the force field was developed, such as traction force microscopy (TFM) and micropillar assay^24–27^. However, due to the random distribution of micro-nano beads in TFM, a particle distribution map without cellular forces is usually required as the reference for data analysis, resulting in the requirement of cell lysis or release and the inability for further tracking or analysis. Moreover, their reliance on a high-magnitude objective lens with a small field of view for imaging greatly restricts their throughput. To eliminate the requirement of reference, periodically and evenly distributed micropillars were developed to replace randomly distributed fluorescent beads. However, due to the limitations of materials and processes, the force-mapping resolution is sacrificed, and downward pressure detection is not possible^28^. In addition, the gaps between the micropillars may disturb cell adhesion formation and maturation^29^. Methods that enable the monitoring of cellular forces in a direct, high-throughput, and low-disturbance manner are needed.

Elastic resonator interference stress microscopy (ERISM) is an impressive approach recently reported that not only eliminates the need for zero-force reference data but also, as a technique for measuring vertical substrate deformation, has successfully observed various cellular mechanical behaviors, such as tumor cell invasion and ameba migration, as well as cellular structures, like podosomes and invadopodia^30,31^. However, tissue level (like monolayer sheets) cellular forces are not possible to acquire due to the need for stress-free regions as boundary conditions, which hinders the possibility of studying cell mechanics behavior across multiple scales^32,33^. Besides, its gold film-coated elastic substrate is distinctly varied from the in vivo biopolymers, which may interfere with the mechanical interactions between cells and substrate^34,35^. Photonic resonator outcoupler microscopy (PROM) utilizes a photonic crystal biosensor to enable dynamic, long-term, quantitative imaging of cell–surface interactions^36^. The PROM technique detects important cellular mechanical structures by detecting changes in the refractive index that result in spectral changes in the photonic crystal reflection spectrum, enabling analysis of cellular mechanics. However, the PROM method does not directly measure cellular forces.

By mimicking the naturally occurring periodic nanostructures in creatures like a chameleon or a butterfly, artificial photonic crystal (PC) materials display brilliant and stable structural colors, showing great potential in chemical, mechanical, and biological signal sensing, and display^37^. Like the skin of a chameleon, when stimulated by an external signal, the periodic nanostructures of PC will be changed, thus altering its photonic stop band through diffraction and interference of light, which in turn alters the reflection spectrum^38–40^. That is, PC materials enable the conversion of the micro-and nano-deformations of periodic nanostructures to the human eye’s perceivable color variations. In light of this, it is plausible to directly obtain local cellular force-related information through the perception of color without any reference or boundary conditions.

Here, by combining a photonic crystal substrate (PCS), which was nano-periodically self-assembled by monodisperse nanoparticles, with a common microscope, we designed a photonic crystal cellular force microscopy (PCCFM) system for in situ cellular force imaging. In addition, a fast algorithm was developed to resolve spectra from a single color image obtained by CMOS to achieve a quantitative analysis of deformations and cellular forces. The PCCFM visualizes and measures cell-induced substrate deformations in sub-micron, allowing for the fast detection of mechanical pathway-relevant sub-cellular structures, like focal adhesions (FAs). The no reference measurement of vertical displacement in a wide-field view makes it possible to track vertically directed cell forces in a high-throughput manner without losing sub-cellular force-mapping resolution (unless otherwise specified, all force-mapping discussed later refers to vertical force-mapping). In addition to the cellular level, PCCFM is also capable of imaging and measuring vertical forces at the tissue level. Together with the stability of PCS, we can monitor real-time cardiac cell monolayer beating and force variations of cell aggregates, suitable for advanced drug evaluation models, like organoids and the organ-on-a-chips. In summary, PCCFM offers a convenient means of obtaining information related to vertical cellular forces, thereby serving as an effective supplement to classical cell force detection techniques, such as TFM.

## Results

### Photonic crystal substrate (PCS) fabrication and characterization

**Fig. 1:**
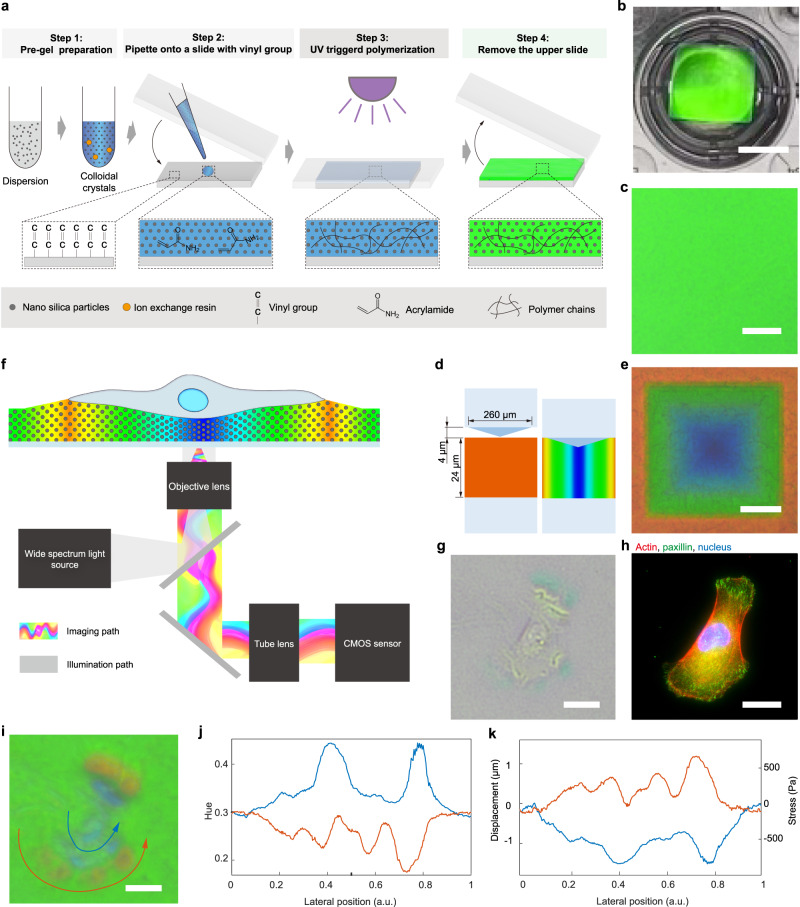
The visualization of vertically directed cell forces by PCCFM system. **a** The fabrication process of PCS. **b** A piece of PCS in a 24-well plate. Scale bar, 5 mm. **c** PCS under ×10 objective lens showed good uniformity. Scale bar, 50 μm. Experiments were repeated five times independently with similar results. **d** The scheme of indenting a 3D-printed pyramidal structure into PCS to produce a color pattern. **e** Image of an orange-red PCS indented by the method in (**d**). Scale bar, 50 μm. Experiments were repeated five times independently with similar results. **f** The scheme of the PCCFM optical imaging system with a wide spectrum light source provides illumination to the PCS and records color light signals carrying deformation information of the PCS. Bright-field (**g**), staining of actin (red), paxillin (green), nucleus(blue) (**h**), and PCCFM (**i**) images of the same MDCK cell. Scale bar, 20 μm. Experiments repeated five times independently with similar results. **j** Two curves of the hue values along the arrow traces in (**i**), and the curve color is the same as the arrow color. **k** Two curves of the vertical displacement and vertical stress along the arrow traces in (**h**), and the curve color is the same as the arrow color.

As a key element in the PCCFM system, a typical PCS is composed of PC film with a thickness of 24 µm and a piece of 1 ⨯ 1 cm^2^ supporting glass, in which silica nanoparticles were periodically distributed in polyacrylamide (PAA) hydrogels with uniform stiffness of 20 kPa (Supplementary Fig. 1). A brief fabrication process is as follows (Fig. 1a, the details of preparation are in the “Methods” section). First, 0.75 g ml^−1^ of 178 nm monodisperse nano-silica particles were well dispersed in the pre-gel solution composed of 4.8% acrylamide (AM), 0.264% N,N’-Methylenebisacrylamide, 5 vol% glycerin, and 2% photoinitiator 2-Hydroxy-4′-(2-hydroxyethoxy)−2-methylpropiophenone. Upon adding the ion exchange resin beads and deionizing the dispersion, colloidal crystals were formed. Then, 2.4 μl of the colloidal crystal was pipetted onto a vinyl-modified 1 ⨯ 1 cm^2^ slide (suitable for a 24-well plate, Supplementary Fig. 2a) and was quickly covered with a normal one to create a chamber with the thickness (*D*) of ≈24 μm to enable self-assembly of nanoparticles. The brilliant color was completely preserved once the pre-gel in the chamber was polymerized by UV light with an intensity of 500 mW cm^−2^ and *λ* of 345–385 nm for 15 s. As a result, the PC film with a stiffness of 20 kPa was covalently attached to the vinyl-modified slide. The fabrication of PCS was completed after removing the covered normal slide. The PCS not only has an eye-visible brilliant structural color (Fig. 1b) but also shows high uniformity under a microscope (Fig. 1c). The rather low hue variation of Fig. 1c (the standard deviation was 0.0025) enables obtaining mechanical signals with a high signal-to-noise ratio. The SEM images of the dehydrated samples of PCS showed their good order and periodicity (Supplementary Fig. 2b–d). When needed, the collagen was covalently linked to the PCS surface by a sulfo-SANPAH crosslinker to enable cell adhesion and growth. Note that, upon requirement, the color can be adjusted by the size and concentration of the silica nanoparticles, and the stiffness can be modified by changing the AM concentration or the UV exposure time, both of which can change the detection range and sensitivity.

### In situ visualization of vertically directed cell force with PCCFM

There is no direct measurement of force in any field of science because Newton’s second law defines force as a quantity that can only be assessed indirectly by assessing the material properties and deformities of physical objects^32^. PCS deformations are directly related to structural color changes, which enables a straightforward way to measure forces. To illustrate this relevance, a pyramid 3D printed structure with a side length of 260 μm and height of 4 μm (Fig. 1d and Supplementary Fig. 3a) was fabricated by two-photon polymerization, which offered submicron force-mapping resolution (Supplementary Fig. 3b)^41^. Afterward, to obtain a full and continuous distribution of structural colors, the pyramid was indented into 4 μm on the orange-red color PCS. Clearly, the deformation degree of the PCS was highly relevant to the structural color (Fig. 1e), illustrating that cellular forces could be captured by the naked eye directly. Together, PCS with periodically distributed monodisperse nano-silica particles have the capacity to directly visualize local cellular forces through color.

Forces generated by cells were imaged with the microscope setup schematically shown in Fig. 1f. Briefly, the PCCFM imaging system can be obtained by minor modification of a common epifluorescence microscope equipped with a color CMOS camera and a wide spectrum tungsten-halogen lamp (see the “Methods” section for the details). We refer to the PCS-based imaging mode as PCCFM mode. When a cell locally deforms the PCS, the periodical structures will be disrupted, resulting in the changing of the reflection spectra, and a color pattern is formed. The color provides an in situ (real-time) measurement of how much deformation the cells have produced in the substrate, allowing for the semi-quantitative measurement of vertically directed cell force magnitude. In this study, to obtain both pulling and pushing induced vertical deformation, the PCS with colors green to yellow (reflection peak *λ* was between 510 and 570 nm) was chosen as it is located in the central region of visible light.

To validate the color pattern of PCS under deformation, a bright-field image (Fig. 1g) and PCCFM image (Fig. 1i) cropped from a 1.3 mm ⨯ 1.0 mm PCCFM image (Supplementary Fig. 3c) of Madin–Darby canine kidney (MDCK) cells cultured on PCS, were obtained after 6 h of seeding. Clearly, the color of the PCS on the outer side of the MDCK lamellipodium changed from green to yellow, i.e., the reflection peak had a redshift, implying the cell-generated upwards pull, while the blue shift in the central region implies the cell-generated downwards push. It means the color pattern could present the semi-quantitative mechanical interaction of MDCK cells with the PCS, rendering researchers to monitor forces in situ without further calculation. Importantly, the deformation profile along the red and blue arrows in the PCCFM image (Fig. 1i) indicates the location and twisting of the focal adhesions (FAs)^30^. To validate the hypothesis, mechanotransduction-relevant elements such as FAs-paxillin and cytoskeleton were analyzed by staining. The yellow region corresponds to the FAs (Fig. 1h), i.e., the PCCFM displayed high force-mapping resolution in the imaging subcellular structure. With the methods mentioned below (Eqs. (1) and (2)), we could calculate the specific vertical displacements and vertical stresses (Fig. 1k) along the arrows from the hue values (Fig. 1j). The maximum upward displacement formed by pulling was around 1.19 μm, while the maximum downward displacement generated by pressing was around 1.52 μm, corresponding to vertical stress of 666 and 848 Pa, respectively (Fig. 1k).

### Vertical micro-deformation measurement with PCCFM

Obtaining high-quality displacement fields exerted by cellular forces is crucial for the calculation of cellular forces and further analysis in mechanobiology^42^. To this end, a simple formula was developed to calculate the vertical deformation ($\Delta D$) from the shift of the peak wavelength (Δ*λ*), the latter of which can be measured directly.

It is thoroughly investigated that the wavelengths of reflection peaks derived by the photonic stop band satisfy the Bragg condition^37,43,44^, $\lambda=2{d}_{111}\sqrt{{n}_{{{\rm {average}}}}^{2}-{\sin }^{2}\theta }$, where ${d}_{111}$ is the interplanar distance of the (111) diffraction planes, ${n}_{{{\rm {average}}}}$ is the average refractive index of the PC, and $\theta$ is the angle between the incident light and the normal to the sample. Here, ${n}_{{{\rm {average}}}}$ and $\theta$ are assumed to remain constant. Thus, a simple formula can be obtained by derivation (in Supplementary Information),

1 $$\frac{\Delta \lambda }{\lambda }=\frac{\Delta D}{D}$$

Briefly, the vertical deformation (Δ*D*) can be deduced from the shift of the peak wavelength (Δ*λ*), in which *λ* and *D* depend on the prepared PCS.

Hyperspectral scanning is a standard method to analyze the change of wavelength (Δ*λ*). However, its relatively slow imaging speed and dependence on specialized equipment might limit its throughput and widespread use^45^. Due to the correlation between the hue and spectra^46,47^, hue-based peak wavelength determination would be an alternative to hyperspectral scanning. To this end, the mapping relationship between hue and reflection peak wavelengths was established for PCCFM.

**Fig. 2:**
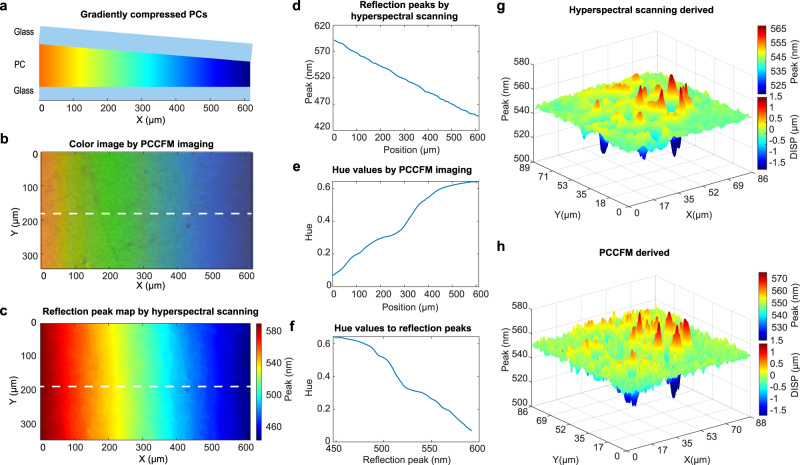
Micro-deformation measurement with PCCFM. **a** The scheme of the gradient squeezing method to generate a color pattern of the PCS. For a better illustration of the relationship between the positions and wavelengths, we define the *x*-axis along the degree of squeezing. **b** The color image captured by PCCFM of the compressed PCS. **c** The reflection peak wavelength map obtained from hyperspectral scanning of the same field of view as in (**b**). **d** The reflection peak wavelengths along the white dashed line in (**c**) as a function of the *x*-position. **e** The hue values along the white dashed line in (**b**) as a function of the *x*-position. **f** The relationship between hue and wavelength is established from (**d**) and (**e**). The reflection peak wavelength map and vertical displacement (DISP) map of the MDCK cell by hyperspectral scanning (**g**) and PCCFM (**h**).

A gradient squeezing method was used to acquire a gradient-deformed PC (Fig. 2a). The generated color pattern was captured by PCCFM for hue information extraction (Fig. 2b), followed by a hyperspectral scan to determine the peak wavelength (Supplementary Fig. 4a). Accordingly, the peak wavelength map along the degree of PCS squeezing was established (Fig. 2c, d). In the meanwhile, by converting RGB values in the color image (Fig. 2b), the point-by-point extracted hue information along the degree of PCS squeezing was constructed (Fig. 2e). Thus, the relationship between hue and reflection peak wavelength can be mapped by pairing their values at the same position (Fig. 2f). Evidently, the hue and the reflection peak wavelength have a one-to-one mapping relationship, demonstrating the reliability of using hue values instead of hyperspectral scans for deformation analysis. Altogether, the vertical deformation (Δ*D*) can be deduced from Eq. (1), in which the peak wavelength variation (Δ*λ*) can be extracted from the hue values in PCCFM images.

To verify the above hue-based micro-deformation measurement method, hyperspectral scanning-derived (Fig. 2g) and PCCFM-derived (Fig. 2h) reflection peak wavelengths of MDCK cells (the PCCFM and bright field images of the MDCK cells shown in Supplementary Fig. 4b, c) were analyzed and compared. Hyperspectral scanning determined longest (pulling forces generated) and shortest (pushing forces generated) reflection peak wavelengths were 570.0 and 511.0 nm, respectively, while those by PCCFM were 577.2 and 514.0 nm, with errors of 1.2% and 0.6%. Typically, the variability in peak wavelength across the field-of-view can be <10 nm with a standard deviation <0.003 (in Supplementary Fig. 4d, e). Unless otherwise specified in subsequent experiments, the wavelengths of the reflection peaks are obtained from this mapping relationship. This reveals that the hue-based PCCFM method can quickly complete high-quality vertical displacement field measurements in a standard biological laboratory, laying the groundwork for the calculation of cellular forces. In addition, since the PCCFM method requires only a single image, it enables measurements to be performed at a high speed, often limited only by the camera frame rate. It is important to note that there should be sufficient crosstalk between different color channels of the camera to ensure a one-to-one correspondence between hue and wavelength, enabling the accurate analysis of narrow-width photonic crystal reflection peaks.

### Vertically directed cell force measurement with PCCFM

The reconstruction of force on a soft elastic substrate requires continuum elasticity theory to describe the deformation of the substrate generated by an external force^42^. Classically, from the displacement field $\mathop{{{{{{\bf{u}}}}}}}\limits^{ \rightharpoonup }$, the Green–Lagrange tensor $\vec{{{{{{\bf{e}}}}}}}$ can be calculated by $\vec{{{{{{\bf{e}}}}}}}=\frac{1}{2}(\nabla \times \mathop{{{{{{\bf{u}}}}}}}\limits^{ \rightharpoonup }+\nabla \times {\mathop{{{{{{\boldsymbol{u}}}}}}}\limits^{ \rightharpoonup }}{\,\!}^{{\rm {T}}})$. In components, this equation reads ${e}_{{ij}}=\frac{1}{2}\times \left(\frac{\partial {u}_{i}}{\partial {x}_{j}}+\frac{\partial {u}_{j}}{\partial {x}_{i}}\right)$. Then, the stress tensor $\vec{{{{{{\boldsymbol{\sigma }}}}}}}$ was obtained as ${\sigma }_{{ij}}=\frac{E}{1+\nu }({\frac{\nu }{1-2\nu }e}_{\alpha \alpha }{\delta }_{{ij}}+{e}_{{ij}})$, which can be simplified to ${\sigma }_{{ij}}=\frac{E}{1+\nu }{e}_{{ij}}$ for incompressible materials. Finally, the stress field $\vec{{{{{{\bf{T}}}}}}}$ can be obtained by ${T}_{i}={n}_{j}{\sigma }_{{ij}}$, where $E$ and $\nu$ represent the two elastic constants of the linear isotropic substrate and ${n}_{j}$ is the normal to the substrate surface.

As the magnitude of each component of a 3D displacement vector was similar^48^, the cellular forces can be characterized by the forces in the vertical direction. Accordingly, the calculation of the stress field $\mathop{{{{{{\bf{T}}}}}}}\limits^{ \rightharpoonup }$ can be simplified as follows and the detailed derivation process is shown in the method. It shows that the magnitude of the local stress $\mathop{{{{{{\bf{T}}}}}}}\limits^{ \rightharpoonup }$ is linearly related to the change in wavelength, with the slope determined by the material’s properties, such as Young’s modulus and Poisson’s ratio. Thus, the forces that MDCK cells exerted on PCS could be quantified after $\Delta \lambda$ (the shift of the peak of the reflection spectrum relative to the substrate in an unstrained state) have been acquired by hue information from a PCCFM image (Fig. 1k).

2 $${T}_{z}={{n}_{z}\sigma }_{{zz}}=\frac{E\times {\varepsilon }_{z}}{1+\nu }=\frac{E}{1+\nu }\times \frac{\Delta \lambda }{\lambda }$$

Overall, PCCFM enables measuring the vertically directed forces through visually perceptible color as well as acquiring the absolute values through low computation.

### High-throughput and time-lapse vertically directed cell force measurement with PCCFM

**Fig. 3:**
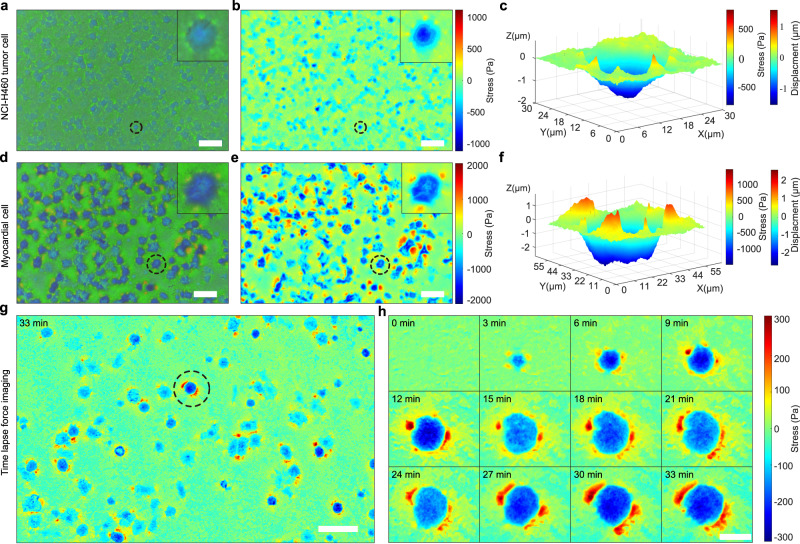
High-throughput and time-lapse vertically directed cell force measurement with PCCFM. PCCFM image **a** and vertical stress map **b** of NCI-H460 cells. Scale bar, 100 μm. Experiments were repeated five times independently with similar results. **c** Vertical deformation profile of the circled region in (**a**) and (**b**). PCCFM image (**d**) and vertical stress map (**e**) of primary myocardial cells. Scale bar, 100 μm. Experiments were repeated five times independently with similar results. **f** Vertical deformation profile of the selected area in (**d**) and (**e**). **g** Vertical stress map of MDA-MB-231 at 33 min after the start of cell attachment. **h** Time-lapse stress map of the selected area in (**g**). Scale bar, 25 μm.

As shown before, we used the PCCFM color pattern image of MDCK to illustrate the capacity of PCCFM in cellular force analysis, which is in situ, low computation, and sensitive in subcellular structures. However, the cellular forces detection-based drug screening method requires high-throughput analysis. In order to achieve this goal, tumor cells and primary cells were used to verify the ability of PCCFM to visualize and measure vertically directed cell forces, strongly relating to total cell forces, in high-throughput, constructing the foundation for later drug screening analysis. PCCFM images and phase-contrast images (Supplementary Fig. 5a) under ×10 magnification of lung tumor cell line-NCI-H460 (Fig. 3a–c) cells and primary myocardial cells (Fig. 3d–f) were acquired after 4 h of seeding on green PCS. Interestingly, a large number of blue spots were observed on the green substrate, showing the blue shift of the wavelength (Fig. 3a). Surprisingly, the edges of the blue spots appeared green to light green changes, which displayed the red-shift (inserted images in Fig. 3a). Clearly, based on the principle of PCS wavelength shift, the pushing force of the lung cancer cells caused a downward deformation of the substrate in the main body of cells, while the pulling of the cells generated an upward deformation of the substrate. It means that without calculation, the vertical force variations under a wide field of view could be acquired by eyes, indicating its application in high-throughput analysis. Based on the correlation between hue information and wavelength shift, as well as Eqs. (1) and (2), the local vertical stress applied on PCS was calculated (Fig. 3b). A representative substrate deformation caused by cellular forces was illustrated (Fig. 3c). Apparently, a 1.524 μm downward deformation and 852 Pa vertical stress in the main body of the cell were exerted on the substrate (Fig. 3c). Notably, at several discrete points around the cell the substrate was deformed significantly (from 0.27 to 0.8 μm) by upward forces (from 151 to 447 Pa), which indicated the presence of FAs. Thus, vertical stress analysis by PCCFM could be achieved under a wide field of view for high throughput without subcellular force-mapping resolution.

PCCFM images of the color pattern generated by primary myocardial cells were also captured and analyzed in the same way (the phase contrast image is shown in Supplementary Fig. 5b). Roughly, compared to that of NCI-H460 (Fig. 3a), a more pronounced regional color change was observed (Fig. 3d), showing the greater forces generated by the myocardial cells than the NCI-H460 cancer cells both in pushing and pulling. To be more detailed, myocardial cells in the circled region showed maximum vertical stress of ~1466 Pa (Fig. 3e), which were almost two-fold of NCI-H460 tumor cells (Fig. 3b). The degree of the upward deformations around the selected myocardial cell (Fig. 3f) was distinctly greater than that of tumor cells (Fig. 3c), indicating their differences in FAs.

In summary, PCCFM demonstrated effective capabilities for visualizing and quantifying vertically directed cell force variations with high-throughput and subcellular force-mapping resolution. This approach offers opportunities for high-throughput drug screening applications.

Due to the stability of the PCS, it is possible to acquire long-term imaging of cellular forces for their dynamic analysis, which is important in processes like tissue folding or tumor migration. To this end, we seeded MDA-MB-231 cells on PCS, and time-lapse images with an interval of 3 min were collected via PCCFM mode under ×10 magnification after 30 min of cell seeding (Fig. 3g, Supplementary Fig. 6, Supplementary Video 1).

To obtain more subcellular information, which is possible in our case and also for better illustration, we cropped the color patterns of one cell (circled in Fig. 3g) at different time points and calculated their vertical stress map (Fig. 3h). Clearly, the cellular vertical stress varied over time and presented a generic gradual rise during spreading (Fig. 3h). Notably, local downward cellular stresses varied from 193 to 289 Pa and upward cellular stresses varied from 116 to 309 Pa over the course of 30 min, indicative of highly dynamic and strong cell–substrate interaction. During the early cell attachment, vertical stress around 100 Pa could also be recorded (Fig. 3h, 3 min), illustrating the high sensitivity of PCCFM in vertically directed cell force detection.

Taken together, the PCCFM demonstrated its powerful ability in visualizing and quantifying the vertical stress of cells from one-shot images or time-lapse imaging sequences in a high-throughput manner. Importantly, it retained the sensitivity and force-mapping resolution, which could provide both cellular and even subcellular level information.

### Monolayer cellular vertical forces measurement with PCCFM

Measurement of myocardial monolayers generated global forces, which is conventionally based on cells in dynamic states (when cells beat), is essential to characterize the effects of drugs and disease on cardiac myocytes and heart function^49–51^. Due to the limitations in references needed for traditional methods, cellular forces in static states that have a significant effect on myocardial tissue have not been thoroughly investigated. Since the PCCFM system requires no references and has a wide field of imaging view as well as high imaging speed, PCCFM is ideal for measuring the full-span activity of myocardial cell sheets, including local and global dynamic and static vertical forces.

**Fig. 4:**
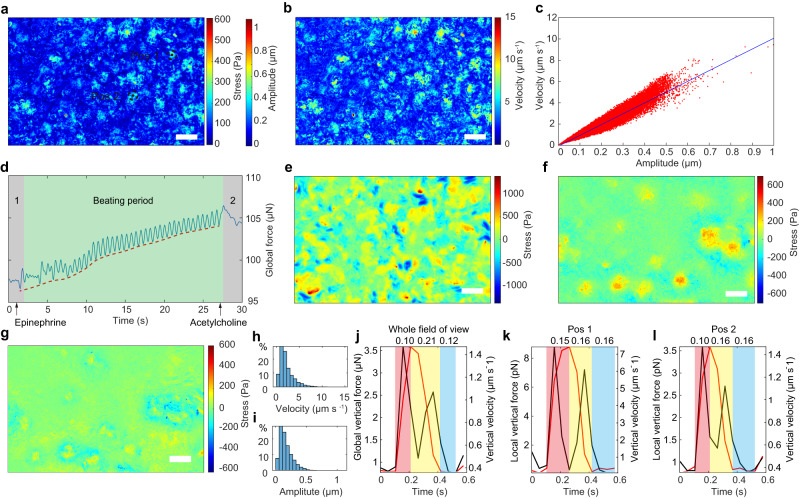
Myocardial cell monolayer vertical force measurement in the PCCFM system. **a** The representative amplitude map and corresponding stress map in one heartbeat period of the myocardial monolayer after 7 days of culture. Scale bar, 120 μm. **b** The corresponding vertical velocity map of **a** by calculating the height difference between two adjacent frames and dividing by 0.051 s. Scale bar, 120 μm. **c** Scatter plot of vertical velocity versus amplitude. **d** Total cellular vertical forces in the field of view over time. The green area represents the beating period, and the gray area represents the static period. **e** The vertical stress map of the beating reduced myocardial monolayer after 14 days of culture. **f** The vertical stress difference between Frame 1 and Frame 2. Scale bar, 120 μm. **g** The vertical stress map eliminated the static stress in the last beat before the addition of acetylcholine. Histogram of vertical amplitude (**h**) and vertical velocity (**i**) in one beating. Total vertical forces and velocity in one beat of the whole field of view (**j**, with an area of 0.61 mm^2^) and of the corresponding position marked in (**a**) (**k**, **l**, with an area of 0.32 μm^2^).

To measure myocardial dynamic states, primary myocardial cells (mainly the ventricular cells) were seeded on PCS for 7 days to generate the myocardial cellular sheet with synchronized beating. Afterward, vertical stress and velocity of myocardial cell sheets were analyzed to characterize their basic function. To this end, a video (Supplementary Video 2) in a random 20 s with 19.06 frames per second was obtained by PCCFM. After the majority of cells in the monolayer sheet had completed one beat, their maximum local vertical stresses and velocities were analyzed (Fig. 4a, b). Evidently, cells in one monolayer displayed various beating behaviors in amplitude, local vertical stress, and velocity. Notably, vertical velocity versus vertical displacement presented a well-linear relationship (*R* = 0.9336) with a slope of 13.99 s^−1^ (Fig. 4c), indicating the maturation of the cardio myocytes^52^.

Myocardial cells sense matrix rigidity through a combination of muscle and non-muscle myosin contractions, which is crucial for many biological processes in health or disease^53^. As the current analysis methods are of low force-mapping resolution in the detection micron level cellular area and reference required, non-muscle myosin contraction stress, which is also called static stress, is quite difficult to analyze in myocardial tissue^54^. In order to track both the dynamic and static stress, non-beating monolayer sheets, which have been cultured for 14 days to vanish the beating (Fig. 4d), were sequentially treated with epinephrine and acetylcholine (Supplementary Video 3). Based on the beating of the myocardial monolayer, we divided the process into static period 1, beating period, and static period 2 (Fig. 4e). Apparently, after the addition of 0.1 nM epinephrine at the first second, the myocardial tissue monolayer rapidly resumed beating with a rise in dynamic stress and reached 99 bpm during 25–27 s. Besides, the lower envelope of the waveform gradually increased (the red dashed line), displaying the enhancement of the static stress. Subsequently, with the addition of acetylcholine at 27th second, the myocardial monolayer stopped beating immediately while the static stress decreased gradually. Interestingly, the static forces in static period 2 are much higher than that of static period 1, indicating the changeable forces in static periods. We used the image at the 1st second (Frame 1, the beginning moment of static period 1) and the image at 28th second (Frame 2, the beginning moment of static period 2) for the calculation of the static stress difference (Fig. 4f). Strikingly, a significant non-uniform distribution was observed, and the areas with increased vertical static stress (green to red or blue areas) are consistent with regions displayed higher vertical stress in the last beating (Fig. 4g). This hysteresis may be caused by the spatiotemporal dependence during mechanotransduction, in which myosin is attached to the stressed cytoskeleton.

Interestingly, in our PCCFM image, cells in one monolayer behaved differently in vertical displacement and velocity (Fig. 4a), which is consistent with the heterogeneities of cellular forces observed by monolayer stress microscopy (MSM)^55^. We performed histogram analysis on all the positions and found that their vertical velocities (Fig. 4h) and displacements (Fig. 4i) were in skewed distribution, indicating cells in one monolayer indeed behaved differently. As PCCFM requires no reference, it can detect both tissue level and cellular level vertical deformation easily, which facilitated us to compare the average vertical forces and velocities generated by the whole myocardial cell sheet in the field of view (Fig. 4j) with those of the local regions (Fig. 4k, l) marked in Fig. 4a in white dashed line circles. The latter is only 1.9 ⨯ 10^−6^ of the whole field of view. In one beating, the duration of their systolic (Red), diastolic (Yellow), and static states (Blue) are different from 0.1 to 0.15, 0.16 to 0.21, and 0.12 to 0.16 s. Moreover, the ratio of maximum systolic to diastolic velocity displayed significant differences, from 0.62 to 0.82 (Fig. 4i). Together, cells in one monolayer have the characteristic of heterogeneity, which may be caused by the complexity of the cellular components, playing an important role in myocardial development and function. This indicates that PCCFM is not only capable of revealing vertical forces at the individual cell level but also allows for high temporal resolution detection at the tissue level, which is quite challenging with current methods.

### Cellular force-relevant pharmacological heterogeneity measurement with PCCFM

Drug resistance and recurrence in tumors are often caused by a relatively small number (such as cancer stem cells) of drug-insensitive cells among the whole cell population^56–58^. As a result, tracking and analyzing the heterogeneity of responses to drugs in cancer cells is imperative^59^. Cellular force is a promising marker to address this, but the current measuring methods suffer from relatively low sensitivity and low throughput^28^. PCCFM can track the heterogeneity of cellular responses to drugs due to its high sensitivity and high throughput capability. Here, by tracking the variation of vertically directed cell forces globally and locally, we screened the heterogeneous responses of MDA-MB-231 breast cancer cells to paclitaxel (PTX), one of the most successful chemotherapeutic drugs for the treatment of breast carcinomas.

**Fig. 5:**
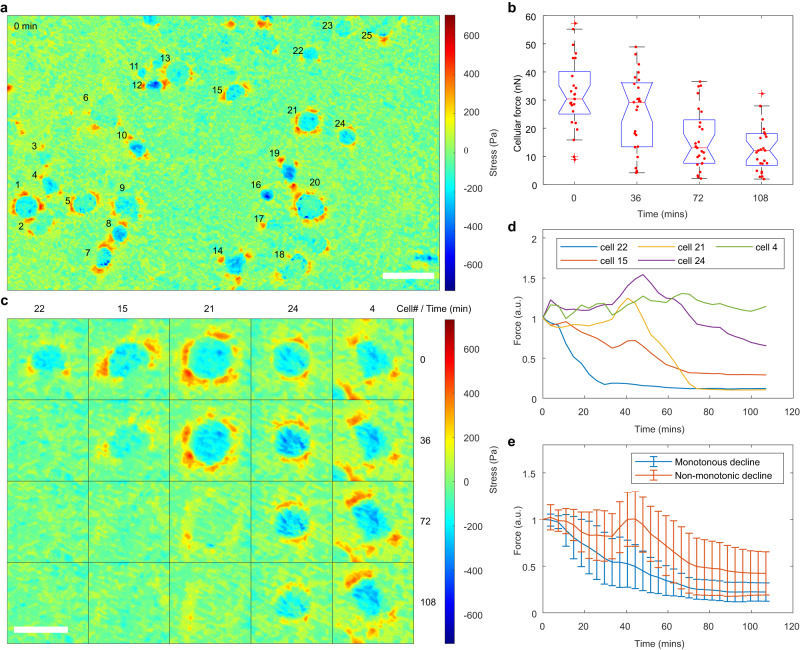
Revealing the heterogeneity of cellular responses to drugs with PCCFM. **a** Vertical stress map of MDA-MB-231 cells treated with PTX at the initial of the experiment. Scale bar, 50 μm. **b** Box whisker plots and scatter plots of the vertically directed cell forces of *n* = 25 MDA-MB-231 cells over time. Experiments were repeated three times independently with similar results. The box represents the interquartile range, with the median depicted by the red line. Whiskers extend to the farthest observations. Outliers beyond this range are marked by red + signs. Notches depict the variability of medians. All data points are marked with red dots. Vertical stress maps (**c**) and the normalized vertically directed cell forces (**d**) of the selected 5 cells over time. Scale bar, 25 μm. **e** The normalized vertically directed cell forces are presented as mean values for two groups of cells over time, with 8 in the monotonically decline group and 16 in the non-monotonically group. Error bars represent the standard deviation (SD).

After MDA-MB-231 breast cancer cells were cultured on PCS for 24 h, they were treated with 50 µM PTX, and immediately phase-contrast images and PCCFM images were captured every 36 min and lasted for about 2 h (Supplementary Fig. 7). In the same way as before, vertically directed cell forces at the four-time points were analyzed (Fig. 5a, b). Clearly, the median of vertically directed cell forces decreased gradually over time, from approximately 30.41 to 12.05 nN, illustrating the validity of using cellular force as a marker in drug screening. Surprisingly, from fast to slow, the vertical stress maps of five representative cells displayed their distinct response rates, indicating different cellular pharmacokinetics to PTX (Fig. 5c, d). It illustrated the meaning of tracking the heterogeneous cell responses to drugs. We also noted a non-monotonic trend that first increased and then decreased (Fig. 5e), despite the overall decreasing trend in vertically directed cell forces (Fig. 5b). This suggests that PCCFM is not only capable of real-time monitoring of drug responses related to cellular mechanics but also enables rapid acquisition of rates and trends, making it an effective tool for the development of mechanically related drugs.

### Vertical force measurement of 3D cell aggregates with PCCFM

2D monolayer and 3D spheroids models have been set up to investigate biophysical and biochemical cues in triggering cell or tissue morphology variations during development or disease generation^60,61^. However, the role of cellular forces in tissue morphogenesis is relatively less underexplored, particularly in 3D models. Limited by force-mapping resolution, throughput, and computational methods, determining the mechanical interactions between cellular forces and substrates inducing cell morphological changes and contributing to tissue morphogenesis in 3D models remains the biggest challenge^62^. As PCCFM is equipped with a large field of view and highly sensitive vertical deformation detection, it allows us to explore the biomechanical information of 3D cell aggregates during morphogenesis. Accordingly, the immortalized human embryonic kidney cells (HEK293V) based 3D cell aggregates were treated with nocodazole, an antineoplastic agent that interferes with microtubules’ polymerization and increases cell contractility^63^.

**Fig. 6:**
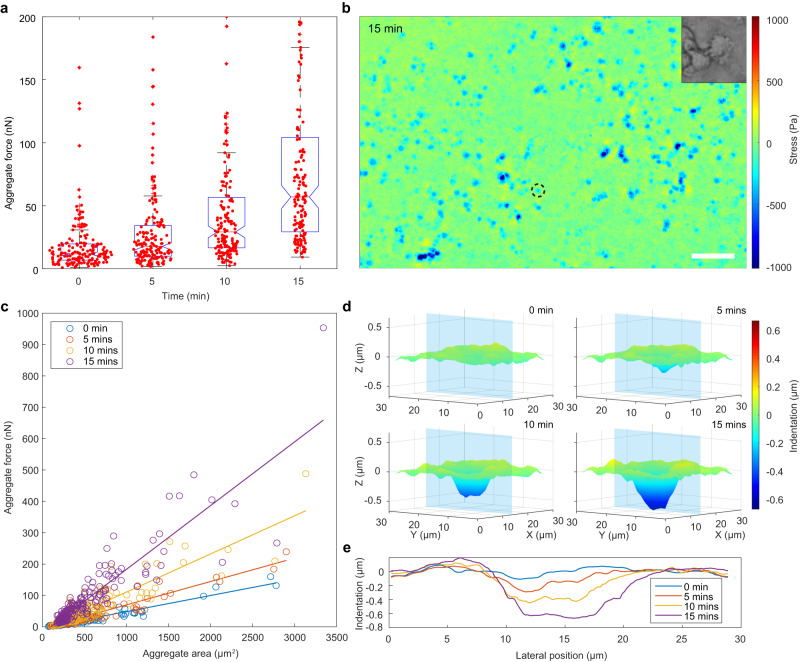
Analysis of vertically directed cell forces of 3D cell aggregates with PCCFM. **a** Box whisker plots and scatter plots of the total vertically directed cell forces generated by *n* = 172 cell aggregates. The box represents the interquartile range, with the median depicted by the red line. Whiskers extend to the farthest observations. Outliers beyond this range are marked by red + signs. Notches depict the variability of medians. All data points are marked with red dots. **b** The stress map of the HEK293 cell aggregates treated with nocodazole at the 15th minute with a bright-field zoom-in of the marked region. Scale bar, 100 μm. A scatter plot of total vertically directed cell forces versus area for individual cell aggregates for four moments was shown in (**c**), and it can be seen that the vertically directed cell forces of the aggregates and the area of the aggregates at the same moment are correlated. Regression analysis was performed on them, and the corresponding regression lines were plotted in the graph. **d** The vertical deformation and stress generated by the cell are marked with a black circle in (**b**). **e** The vertical displacement at the cross-section is caused by the indentation of the cell aggregate in (**d**) at four moments.

After HEK293V cells were cultured on the PCS for 24 h, they were treated with 5 nM nocodazole and then immediately imaged every 5 min and lasted for about 15 min (Supplementary Fig. 8). Over the course of 15 min, vertical forces exerted by the cell aggregates on the substrate was rapidly and significantly enhanced (Fig. 6a and Supplementary Fig. 8), consistent with trends based on discrete cells^64^. The color pattern of the substrate at 15 min displayed the maximum squeeze stress of 1.27 kPa (Fig. 6b). Notably, most cell aggregates conformed to a fitting curve at each time point, and the slope of the curve increased from 0.05 to 0.20 nN/μm^2^ over time (Fig. 6c). In other words, the increasing of compression was faster than that of the area. Contrary to the small increase in area from 2781 to 3343 μm^2^, a 4.97-fold increase in compression forces from 159.63 to 953.30 nN was observed, demonstrating the importance of vertically directed cell forces tracking during 3D morphogenesis. In particular, the horizontal area of interaction between the aggregates and the substrate did not change significantly, but the vertical area showed a downward displacement of the inner region and an upward displacement of the outer edge (Fig. 6e). According to the surface information of the blue section position in Fig. 6d, we found that the pushing depth increased from 0.11 to 0.66 μm, and the pulling height increased from 0.09 to 0.19 μm, displaying a V-shaped push on the substrate (Fig. 6e).

By detecting and measuring the response pattern of HEK293 3D aggregates to nocodazole, PCCFM demonstrates its ability to monitor cell–substrate biomechanical interactions in 3D tissue, implying their potential use to investigate cellular forces variation in 3D tissue morphogenesis and tumorigenesis.

## Discussion

We presented a photonic crystal-based method for cellular force imaging that makes use of PC’s diffraction and interference of visible light to visualize and quantify vertically directed cell forces through color changes. The above examples illustrate how PCCFM visualizes and measures it on the PCS substrate in an in situ, high-throughput, and cross-scale manner.

The PCS used in the PCCFM displayed excellent capability in converting cell-generated vertical deformations to eye-readable color variations, making it possible to monitor vertically directed cell forces in situ. Moreover, we can visualize the vertical forces without calculation and other end-point experiments, which is quite friendly to researchers. When capturing images with a ×10 objective lens, it is already sufficient to detect color differences generated by subcellular structures, thus providing the possibility for high-throughput analysis. In case a higher accuracy (and/or a lower background) is required, pre-measurements of the substrate’s texture before cell seeding can be performed to eliminate background noise arising from variability in the reflection peaks. The noise and truncation errors in the CMOS camera can cause discrepancies in hue analysis. Utilizing a low-noise and high-bit-depth camera can help mitigate such errors. Moreover, these types of noise can be suppressed through the implementation of algorithms, such as temporal and spatial filtering, further improving the accuracy of hue analysis. Theoretically, in a well-calibrated system, detection can be achieved using just two channels, attaining a satisfactory signal-to-noise ratio within specific spectral ranges. However, this approach is relatively more susceptible to interference from noise and background light intensity. In our PCCFM system, a robust but low computational calculation model is developed to quantify the vertically directed cell forces strongly related to total forces in magnitude. Notably, high-quality vertical displacements can be calculated from the color changes in the PCS without the need for reference or additional operations in the measurement process. This feature enables the application of PCCFM in drug screening in the example of PTX treatment to MDA-MB-231 cells. By high-throughput analysis of cellular forces, differences in response trends and rates were observed, illustrating the importance of tracking heterogeneous responses to drugs. The no-reference feature of PCCFM and the stability of the PCS facilitated the long-term cellular forces measurement over 14 days.

The intrinsic sensitivity of the periodical arrangement of nanoparticles to external stimuli in PCS determines their sensitivity in sensing vertically directed cell forces. A weak cellular force will induce the displacement and color variations of PCS, which renders PCCFM particularly suited for measuring vertical deformations with high sensitivity. Moreover, the densely arranged nanoparticles (around 2000 nanoparticles per cell, 20 per square micrometer) facilitate the PCCFM to detect cellular forces in a small area and provide subcellular mechanical information, like FAs. Importantly, high throughput and high force-mapping resolution are normally not compatible with one device. However, in our system, the features of PCS enable us to obtain the subcellular, cellular, and tissue-level vertical force information via one image. For example, biomechanical details like FAs are barely observed under the bright field but could be in situ sensed by PCCFM without traditional immunofluorescence staining. Compared with TFM, the pillar array can measure cell forces at the subcellular level only when the pillars are close enough, which makes the preparation of such a substrate not easy in the laboratory^28,32^.

Functional and pathological changes in cells are often accompanied by changes in cellular forces as well, like the drug resistance achieved by breast cancer cells through mitochondrial fission related to the contracting forces, the activation intensity of cytotoxic T cells and B cells directly related to the cell forces, and the change of cytoskeleton during the release of viruses from cells can be directly reflected by cellular forces, making the cellular force-based drug screening have a broader application, no matter it is oncology drug discovery or vaccine development. In recent years, advances in high-throughput cell force measurement techniques for drug screening have been achieved to some extent. For example, fluorescently labeled elastomeric contractible surfaces (FLECS) achieve 100-fold greater throughput in measuring single-cell forces than the existing single-cell tools, which can be used to test customized asthma medications. However, it lacks subcellular force-mapping resolution. Since PCCFM has the characteristics of a large field of view, high speed, and no reference needed, it not only provides a comparable high throughput to FLECS but also a higher force-mapping resolution for better drug screening. PCCFM shows advantages in sensing both tumor cell line NCI-H460 exerted weak vertical forces and primary neonatal mouse cardiomyocytes generated strong vertical forces, demonstrating the applicability of PCCFM in cellular force-relevant drug screening. Besides, their significant differences in the color patterns provided a possibility for cell sorting.

Dynamic stress analysis of myocardial cell monolayer is a mature model for the research of cardiac development and cardiotoxicity assay^51^. Since PCCFM is equipped with no-reference and high-speed features, it has allowed the static and dynamic stresses of a myocardial cell monolayer to be measured simultaneously, and with spatiotemporal force-mapping resolution. This offers the possibility to better investigate static stress-induced myocardial tissue maturation and pathophysiology. Based on the real-time vertical deformation recording, analyses like vertical beating amplitudes, velocities, and heart rates were readily calculated to evaluate the developmental process and functional changes of myocardial tissue. Moreover, the differences between the global and the local stress illustrated the importance of revealing subtle alterations in large fields of view as a complement to the current evaluation method, which may be masked by averaging. The global and local vertical force analysis of monolayer sheets under a ×10 objective also certificated the powerful capacity of PCCFM in imaging both the cellular level and tissue level forces.

Tissue-level cellular forces analysis remains challenging, particularly in developmental and pathological studies. Compared with much fewer morphological variations, the downward forces of the tissue-level HEK293V cell aggregates raised significantly in one experiment, implying the importance of tissue-level vertical force detection thanks to the large field of view of PCCFM. The tissue level force variations may activate local force-sensitive proteins, alter cell or tissue morphologies, and eventually induce tissue morphogenesis and tumorigenesis. For example, neural tube organoids have been constructed in different ways^65,66^, but the folding process, which involves force variation and morphology change, remains to be explored. PCCFM, which enables tissue-level vertical force detection, is promising in characterizing organoid interface behavior.

The elements (nanoparticles and gels) to prepare PCS are not only easily accessible and low-cost but also versatile, making it possible to fabricate specific substrates based on the context of the usage. Hydrogel-based PCS provides the possibility to fabricate substrates closer to in vivo microenvironment, not only with different stiffness to fit different types of in vivo tissues but also with non-linear mechanical properties such as stress-relaxation or stress-stiffening. Besides, magnetic responsive nanoparticles can also be applied, which will provide additional mechanical stimuli. Although PCCFM enables the vertical force measurement of a 3D cell complex via 2D culture, it is not available for 3D measurement currently. Special PCS that can accomplish high-quality self-assembly in an environment where cells and buffers are present. Besides, PCCFM is not applicable to in vivo measurement currently.

It is worth noting that PCCFM could serve as a complementary technique to TFM, with advantages in measuring substrate thickness changes induced by cells. While PCCFM allows for the measurement of vertically oriented cell forces, it does not directly replace TFM in providing comprehensive mechanical information (Supplementary Fig. 9). Furthermore, due to some approximations in the hue variation and deformation model, the results obtained by PCCFM may be less accurate than TFM in certain situations.

In summary, we developed a vertically directed cell force measurement system, which is in situ, with high throughput and high sensitivity, and cross-scale. It enables the visualization and measurement of vertical cell forces in multiple cellular complexes from a 2D single cell to 3D cell aggregates, providing cellular force information from the subcellular level, cellular level to tissue level. It may provide a tool for cellular force-relevant drug screening and give insights into cell mechanics in tissue morphogenesis and tumorigenesis in a more real-time and large-scale manner.

## Methods

### Photonic crystal substrate fabrication

The photonic crystal substrate (PCS) is composed of periodically arranged silica nanoparticles and polyacrylamide hydrogels. The pre-gel dispersion was composed of 0.75 g ml^−1^, 178 nm silica nanoparticles (monodisperse silica nanoparticles with sulfonic acid groups, Avatarget Suzhou), 4.8% acrylamide (AM), 0.264% N,N’-methylenebisacrylamide (BIS-AM), 5 vol% glycerin, and 2% photoinitiator 2-hydroxy-4′-(2-hydroxyethoxy)-2-methylpropiophenone. In this colloidal mixture, AM acts as a monomer of the hydrogel, and BIS-AM as a crosslinker. Glycerin was blended to modulate the dispersion force and promote successful separation from the template. To achieve well-dispersed monodisperse silica nanoparticles in the pre-gel, the mixture was treated with vigorous stirring and ultrasonication. 10 ion exchange resin beads (Bio-Rad’s AG 501-X8 Mixed Bed Resin, biotechnology grade, catalog number #1437424) were added into the dispersion using tweezers to deionize the dispersion for the colloidal crystals formation. The crystal formation process typically took <5 min. Because of the repulsive forces between silica nanoparticles, they self-assembled into non-close-packed colloidal crystals in the dispersion. Afterward, 2.4 μl of the pre-gel was added onto a piece of a vinyl-modified glass slide with the size of 1 ⨯ 1 cm^2^ and then gently covered with another normal glass slide to form a chamber with a thickness of ≈24 μm. The thickness of the chamber can be well controlled by adjusting the amount of pre-gel and the size of the slide. According to Eqs. (1) and (2), it is known that the sensitivity and thickness are inversely proportional, so the sensitivity of the substrate can be improved by reducing the thickness. By slightly kicking the slides on the bench, the pre-gel was sheared to promote the monocrystallization of the colloidal crystals. The brilliant colors were maintained once the pre-gel suspension in the chamber was exposed to UV light with an intensity of 500 mW cm^−2^ and wavelengths of 345–385 nm for 15 s. The photonic crystal film was readily left on the vinyl-modified side of the glass once the gel was formed. After removing the upper slide, the PCS was obtained, which can be stored in water or phosphate-buffered saline (PBS, Biosharp, cat. no. BL302A) for several weeks in the fridge.

### Nanoindenter

Substrate stiffness was measured using a nanoindenter (Piuma Nanoindenter, Optics11life). The stiffness (*k*) of the probe was 3.160 (N m^−1^), and the tip radius (*R*) was 51.5 μm. The Hertzian contact model was used to calculate the effective Young’s modulus.

### PCS modification by Collagen Type I

For cell culturing, the surface of PCS was functionalized by rat tail type I collagen using Sulfo-SANPAH. Sulfo-SANPAH crosslinker (N-Sulfosuccinimidyl-6-[4’-azido-2’-nitrophenylamino] hexanoate) is a water-soluble protein crosslinker. The Sulfo-SANPAH crosslinking reagent contains an NHS ester that reacts with primary amines and a nitrophenyl azide group that is photoreactive toward amino groups. After aspirating the water from the surface of the PCS substrate, 50 μl of 2 mg ml^−1^ Sulfo-SANPAH solution was added to completely cover the surface of the PCS. Then, the PCS was treated by a UV light with an intensity of 50 mW and *λ* of 345–385 nm for 10 min. After the PCS was rinsed with PBS (Biosharp, cat. no. BL302A) to remove excess sulfo-SANPAH, 50 µl of 0.15 mg ml^−1^ Collagen Type I solution was pipetted onto the surface of the PCS and left at 4 °C overnight for cross-linking. Before cell seeding, the PCS was rinsed with PBS (Biosharp, cat. no. BL302A) 3 times to remove excess collagen solution and sterilized under UV (*λ* = 254 nm) for 30 min. The sterilized PCS can be used for cell seeding immediately or stored in the fridge for several weeks. Generally, PCS was placed in a 24-well plate for cell culture with 1.5 ml of medium. The cell seeding density depends on each cell type. After the desired cell suspension was acquired, they were added on top of the PCS for adhering and later cellular force tracking and analysis.

### PCCFM imaging system

The PCCFM imaging system was obtained by minor modification of a commercial inverted epifluorescence microscope (ECLIPSE Ti2, Nikon) equipped with a color CMOS camera (DS-Ri2, Nikon) and a tungsten-halogen lamp. The field aperture of Köhler illumination optics was turned smaller to achieve high contrast and low background during the measurement. A 50/50 beam splitter was set in the fluorescence cube without any filter. When imaging a PCS, broad-spectrum light (400–1000 nm) from a tungsten halogen lamp was reflected by the beam splitter and projected onto the PCS by a ×10 objective lens (N.A. 0.3, Nikon). Then, the PCS assigns local deformation information to the reflected light. A color CMOS camera then imaged the colored view to obtain a stress map directly. The measurement speed was 30 frames per second in the current system. Further quantitative analysis can be obtained algorithmically from these color images. As the color sensing of the camera is affected by tungsten-halogen lamp intensity, camera sensitivity settings, objectives, and white balance settings, these parameters need to be kept constant during PCCFM imaging. Bright-field imaging images could be directly obtained by closing the light shutter of the above broad-spectrum illumination and using the transmitted light source.

### PCCFM data analysis

After the PCCFM images were captured, the hue values were extracted from the obtained images pixel by pixel, and then the wavelengths corresponding to these hue values were obtained by looking up the table based on the curves in Fig.2g. Then, the deformation map at each pixel position obtained according to Eq. (1) and the stress map could be calculated by Eq. (2). The relevant parameters are as follows; Young’s modulus ${E}={20}\,{{{{{\rm{kPa}}}}}}$, Poisson’s ratio ${\nu }={0.49}$.

The local vertical force was calculated by adding the forces at each pixel of each region using the equation ${{F}}_{{\rm {{local}}}}=\sum {T}_{z}\times {{{{{{\rm{length}}}}}}}_{{{\rm {pixle}}}}^{2}$. The length of pixel for DS-Ri2 camera and the ×10 objective was 0.18 μm for full resolution. The global vertical force was calculated by summing up the forces for each pixel. The global average vertical force was obtained by dividing the global vertical force by the total area.

### Hyperspectral scanning

The homemade hyperspectral scanning system consisted of a commercial inverted epifluorescence microscope base (ECLIPSE Ti2, Nikon) equipped with a tungsten-halogen lamp as a broad-spectrum light source, a liquid crystal tunable filter (LCTF, KURIOS-VB1, THORLABS), and an sCMOS camera (PCO.edge 4.2 bi). During epi-illumination, light from the tungsten-halogen lamp passed through liquid light guide fibers and then was focused onto the sample by the objective. Using the same objective, the backscattered light was collected, passed through the LCTF, and imaged by the sCMOS camera. Multiple wide-field monochromatic images are obtained for each acquisition and stored in an image stack. LCTF was set to narrowband mode. Multiple backscattered wide-field monochromatic images were obtained across a range of wavelengths (420–730 nm, step size 1 nm) to produce a hyperspectral image stack, *I*(*λ*, *x*, *y*), where *λ* represented the wavelength and (*x*, *y*) corresponded to the pixel position. The exposure time for each image was 50 ms. Then we use the equation ${{I}}_{{\rm {P{C}}}_{{\rm {{reflection}}}}}\left(:,{x},{y}\right)={I}\left(:,{x},{y}\right)./{{I}}_{{\rm {{bg}}}}-1$ to obtain the reflection spectrum of the corresponding point PCS. Where ${{I}}_{{\rm {P{C}}}_{{\rm {{reflection}}}}}$ denotes the reflection spectrum of the PCS at the $\left({x},{y}\right)$ position, ${I}\left(:,{x},{y}\right)$ denotes the spectrum detected at the (*x*, *y*) position, and ${{I}}_{{\rm {{bg}}}}$ denotes the background spectrum.

### Two-photon laser polymerization printing

A four-sided pyramid with a side length of 240 µm, a height of 4 µm, and a layer thickness of 250 nm was fabricated on a glass substrate by two-photon polymerization laser direct writing technology and characterized by SEM. The pyramid was fabricated using a commercial two-photon lithography system (Photonic Professional GT, Nanoscribe GmbH) equipped with a fiber laser operating at a wavelength of 780 nm, a pulse frequency of 80 MHz, and a pulse duration of 120 fs. An oil immersion objective (×25, NA = 1.4, WD = 190 µm) was used. The fabrication was carried out by first loading a drop of the resin (IP-S, Nanoscribe GmbH) onto a clean coverslip. Then, the coverslip was placed on the Nanoscribe stage for two-photon processing. Following laser exposure, the sample was developed by soaking first in propylene glycol monomethyl ether acetate (Sigma-Aldrich) for 15 min and then in 2-propanol (Sigma-Aldrich) for 2 min. Finally, the sample was dried with nitrogen gas. Both resin preparation and composite fabrication were carried out in a UV-free environment. All chemicals used in the current study were used as received.

### Critical point drying for SEM

PCS with cells were fixed in 3% precooled glutaraldehyde for 120 min at 4 °C and then rinsed twice with PBS (Biosharp, cat. no. BL302A) for 10 min each. Following sequential dehydration in an ethanol gradient (30%, 50%, 70%, 80%, 90%, 95%, 100%, 15 min for each concentration), fixed hydrogels with cells were air-dried overnight and dried using critical point drying.

### Cell culture for forces analysis

Madin–Darby canine kidney (MDCK) cells were bought from National Collection of Authenticated Cell Cultures and cultured in DMEM medium supplemented with 10% fetal bovine serum (FBS, Gibco), 2 mM glutamine (Sigma-Aldrich, cat. no. 1294808-100MG), and 1% Penicillin–Streptomycin (P/S, Gibco, cat. no. 15140122). Cells were cultured in an incubator at 37 °C with 5% CO_2_. After MDCK cells were 80–90% confluent, cells were dissociated, resuspended, and seeded on the PCS with a density of 10,000 cells per square centimeter. After being inoculated on the PCS for 4 h, they were placed under the PCCFM for imaging. Immediately following imaging, they were stained with vinculin for FAs analysis. Firstly, they were washed with PBS (Biosharp, cat. no. BL302A) and fixed directly on the PCS with neutral buffered 10% formalin solution (Solarbio, cat. no. G2161) at room temperature for 10 min. Following, it was washed with 0.05% Tween 20 (Sigma-Aldrich, cat. no. P1379-25ML) in PBS (Biosharp, cat. no. BL302A), permeabilized with 0.1% Triton X-100 (Sigma-Aldrich, cat. no. T8787-50ML) for 3 min, and blocked with 1% BSA (Sigma-Aldrich, SRE0096-10G) in PBS (Biosharp, cat. no. BL302A) for 30 min. Cells were then stained for paxillin using a rabbit anti-paxillin rabbit monoclonal (Abcam, cat. no. ab32084, 1:100 in BSA solution) at 4 °C overnight, followed by the secondary goat anti-rabbit FITC-conjugated antibody (Sigma-Aldrich, 1:32 in BSA solution) for 2 h at room temperature. Actin was stained in parallel with the secondary antibody by using 1:100 diluted Actin-Tracker Red solution (Beyotime Biotechnology, cat. no. C2203S). Cell nuclei were stained with DAPI (Merck Millipore, 1:1000 in BSA) for 3 min at room temperature.

### Vertically directed cell force analysis of NCI-H460 cells

NCI-H460 cells were bought from the National Collection of Authenticated Cell Cultures and cultured using RPMI-1640 medium (Gibco, cat. no. 11875093) supplemented with 10% FBS (Gibco) and 1% P/S solution (Gibco, cat. no. 15140122) in a 25 ml cell culture flask. A cell density of 30,000 cells per square centimeter was seeded on PCS. After 4 h of inoculation, they were imaged directly under PCCFM for high-throughput analysis.

### Vertically directed cell force analysis for myocardial cells

First, primary neonatal rat myocardial cells were isolated from 1-day Sprague-Dawley rats of both sexes. In brief, neonatal rat ventricles were digested and purified with Collagenase I (Sigma-Aldrich, cat. no. C0130-100MG) by 1 h incubation at 37 °C in a 5% CO_2_ incubator. The collected cardiomyocytes were cultured in DMEM (Gibco, 21013024) with 10% FBS (Gibco) in a flask. The cell culture medium was refreshed every 2 days. All procedures involving animals were conducted in accordance with the Guide for the Care and Use of Laboratory Animals published by the US National Institutes of Health (NIH Publication no. 85-23, revised 1996) and approved by the Southeast University Committee on Animal Care.

For a high-throughput vertically directed cell force imaging experiment, the primary myocardial cells were seeded with a cell density of 20,000 cells per square centimeter on PCS and imaged directly under PCCFM after 4 h of seeding.

For myocardial cell sheet dynamic force analysis, the primary myocardial cells were seeded at a density of 30,000 cells per square centimeter. Then, the cell culture medium was refreshed every day for 7 days. Afterward, they were imaged directly under PCCFM for beating analysis.

For myocardial cell sheet dynamic and static vertically directed cell force analysis, the primary myocardial cells were seeded at a density of 30,000 cells per square centimeter. The cell culture medium was refreshed every day for 14 days. Then the plate with PCS and cells were placed under the PCCFM microscope, which was in a stage-top incubator with a steady CO_2_ and temperature control. Once the experiment started, video recording in PCCFM mode was performed using the camera of the microscope. A working concentration of 0.1 nM epinephrine (Aladdin, cat. no. L137183) was added at the 6th second. Then A working concentration of 100 nM acetylcholine (Aladdin, cat. no. A111015-25g) was added at the 34th second.

### Vertically directed cell force analysis for MDA-MB-231 cells

MDA-MB-231 breast cancer cells were bought from the National Collection of Authenticated Cell Cultures and cultured using L-15 medium (Gibco, cat. no. 11415064) supplemented with 10% FBS (Gibco) and 1% P/S solution (Gibco, cat. no. 15140122) in a 25 mL cell culture flask. Cells were cultured in an incubator at 37 °C with 5% CO_2_. For real-time cellular force imaging experiment, images were recorded after 30 mins of adhesion with a density of 10,000 cells per square centimeter. MDA-MB-231 cells were seeded at a density of 10,000 cells per square centimeter for 24 h before drug treatment. Afterward, a working concentration of 50 µM nocodazole (Aladdin, cat. no. P106868-10mg) was added. Then the plate with PCS and cells was placed under the PCCFM microscope, which was in a stage-top incubator with a steady CO_2_ and temperature control.

### Vertically directed cell force analysis for cell aggregates

HEK293V cells were bought from the National Collection of Authenticated Cell Cultures and cultured in DMEM (Gibco, 21013024) supplemented with 10% FBS (Gibco) and 1% P/S solution (Gibco, cat. no. 15140122) in a T25 cell culture flask. HEK293V cells were seeded at a density of 30,000 cells per square centimeter for 24 h before drug treatment. Afterward, a working concentration of 5 nM nocodazole (Aladdin, cat. no. N408505-1ml, dissolved at 10 mM in DMSO) was added. Then the plate with PCS and cells were placed under a Ti2 microscope (Nikon), which was in a stage-top incubator with a steady CO_2_ and temperature control.

### Reporting summary

Further information on research design is available in the Nature Portfolio Reporting Summary linked to this article.

### Supplementary information

Supplementary Information

Reporting Summary

Description of Additional Supplementary Files

Supplementary Movie 1

Supplementary Movie 2

Supplementary Movie 3

## Data Availability

The PCCFM code (including algorithms for calculating the peak wavelength of reflections) and example images can be accessed at 10.5281/zenodo.8419840. The code is specifically designed for analyzing PCCFM images and comes with comprehensive documentation. Any additional data related to this study are available from the corresponding author upon request.
