# Supplementary material for: Imaging cellular forces with photonic crystals
Source: Nat Commun. 2023 Nov 14;14:7369. doi: 10.1038/s41467-023-43090-9 (PMC10646022; doi:10.1038/s41467-023-43090-9)
Supplement: Supplementary file 1 — Supplementary Information [file 41467_2023_43090_MOESM1_ESM.pdf]

Supplementary Materials:

Imaging cellular forces with photonic crystals

Qiwei Li[1]†, Zaozao Chen[1,2]†, Ying Zhang[1]†, Shuang Ding[1], Haibo Ding[1], Luping Wang[1,3], Zhuoying Xie[1], Yifu Fu[1], Mengxiao Wei[1], Shengnan Liu[1], Jialun Chen[1], Xuan Wang[1] and Zhongze Gu[1,2]*

[1] State Key Laboratory of Bioelectronics, School of Biological Science and Medical Engineering, Southeast University; Nanjing, Jiangsu, 210096, China.

[2] Institute of Biomaterials and Medical Devices, Southeast University; Suzhou, Jiangsu, 215163, China.

[3] Faculty of Sports Science, Ningbo University, Ningbo 315211, China.

*Corresponding author. Email: gu@seu.edu.cn

† These authors contributed equally to this work.

Deriving the relationship between $\Delta\lambda$ and ΔD from the Bragg condition

Since photonic crystals have a nanoscale periodic structure and interact strongly with light through Bragg diffraction, they are an ideal substrate material. To model the process, Maxwell's function is used and can be simplified to

$$\nabla \times (\frac{1}{\varepsilon(\vec{\boldsymbol{r}})}\nabla \times \vec{\boldsymbol{H}}(\vec{\boldsymbol{r}})) = (\frac{\omega}{c})^2\vec{\boldsymbol{H}}(\vec{\boldsymbol{r}}) \quad (1)$$

$$\varepsilon(\vec{\boldsymbol{r}}) = \varepsilon(\vec{\boldsymbol{r}} + \vec{\boldsymbol{a}}) \quad (2)$$

where $\vec{\boldsymbol{H}}$ is the magnetic field strength of the electromagnetic wave, $\vec{\boldsymbol{a}}$ is the period of the dielectric coefficient represented by $\varepsilon(\vec{\boldsymbol{r}})$, and ω is the angular frequency of the light. Then, the photonic band structure can be obtained in the Brillouin zone using Bloch's theorem and numerical calculation methods, indicating the intensity of light reflected by Bragg's diffraction. The band structure of the face-centered cubic structure, a single-peaked stop band when incident perpendicular to the (111) diffraction plane, has long been thoroughly investigated. Therefore, the peak center of the reflection spectrum should satisfy the Bragg condition[46,47].

$$\lambda = 2d_{111}\sqrt{n_{average}^2 - sin^2\theta} \quad (3)$$

where d_{111} is the interplanar distance of the (111) diffraction planes, $n_{average}$ is the average refractive index of the photonic crystal, and θ is the angle between the incident light and the normal to the sample. Here, $n_{average}$ and θremain constant during the experiment.

The thickness of the photonic crystal hydrogel (*D*) was equal to

$$D = Md_{111} \quad (4)$$

where M is the total number of layers of the (111) diffraction planes.

When an external force causes deformation of the regional hydrogel substrate, there is a change in the thickness *D*. Thus, the shift of the peak wavelength can be deduced from the ratio of thickness change.

$$\frac{\Delta\lambda}{\lambda} = \frac{\Delta\ (\frac{M\lambda}{2\sqrt{n_{average}^2 - sin^2\theta}})}{\frac{M\lambda}{2\sqrt{n_{average}^2 - sin^2\theta}}} = \frac{\Delta D}{D} \quad (5)$$

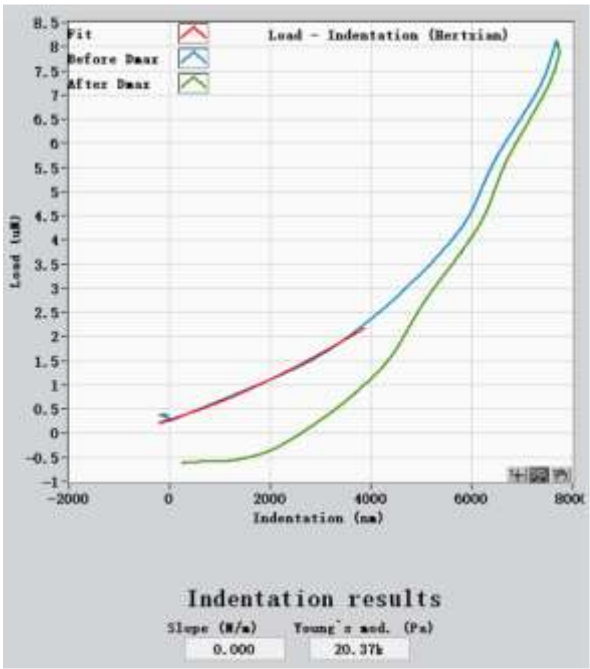

Supplementary Figure 1 Characterization of photonic crystal substrate (PCS) stiffness with Piuma Nanoindenter, Optics11life. The load-indentation curves were obtained from the nanoindentation test. The green curve was recorded during loading, and the blue curve was recorded during unloading. The red curve was obtained by fitting the green curve with the Hertzian contact model by Piuma software, and Young's module obtained was 20.37 kPa.

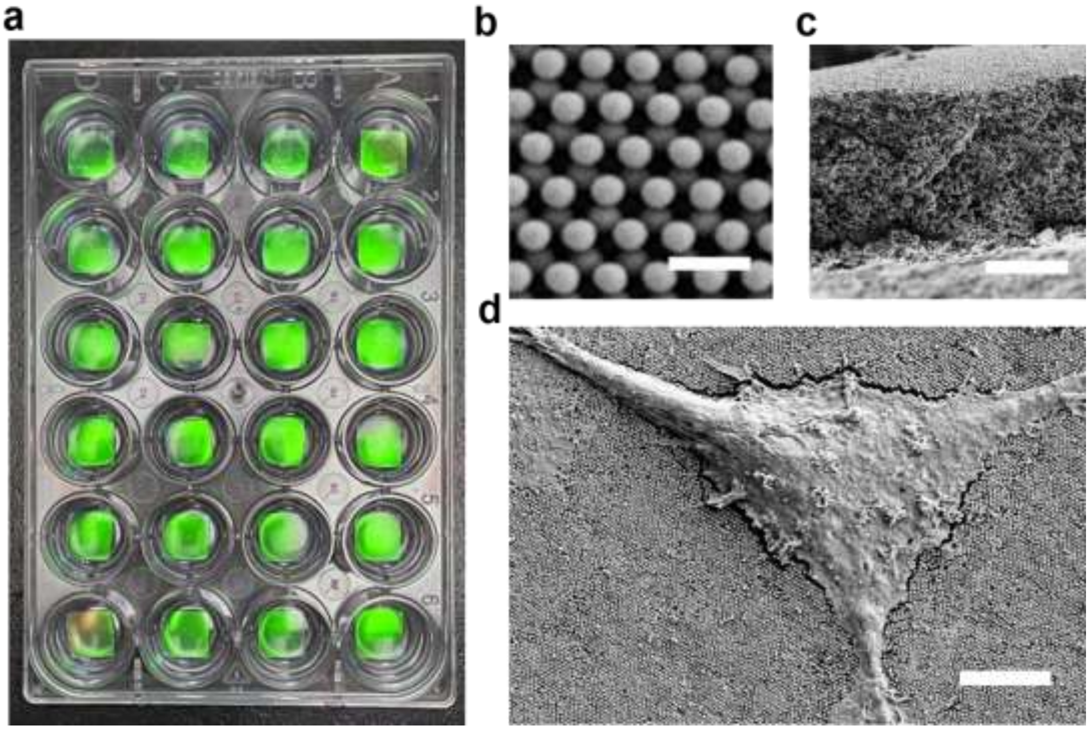

Supplementary Figure 2 A macroscopic photograph and microscopic scanning electron microscopy (SEM) images of PCSs. (a) Scale-up prepared PCS were placed in 24-well plates for high-throughput drug screening. **(b)** Top view of the dehydrated PCS with SEM. Scale bar, 450 nm. **(c)** Cross section of the dehydrated PCS with SEM. Scale bar, 4 μm. (**d**) SEM image of a dehydrated

cell grown on PCS. Scale bar, 5 μm.

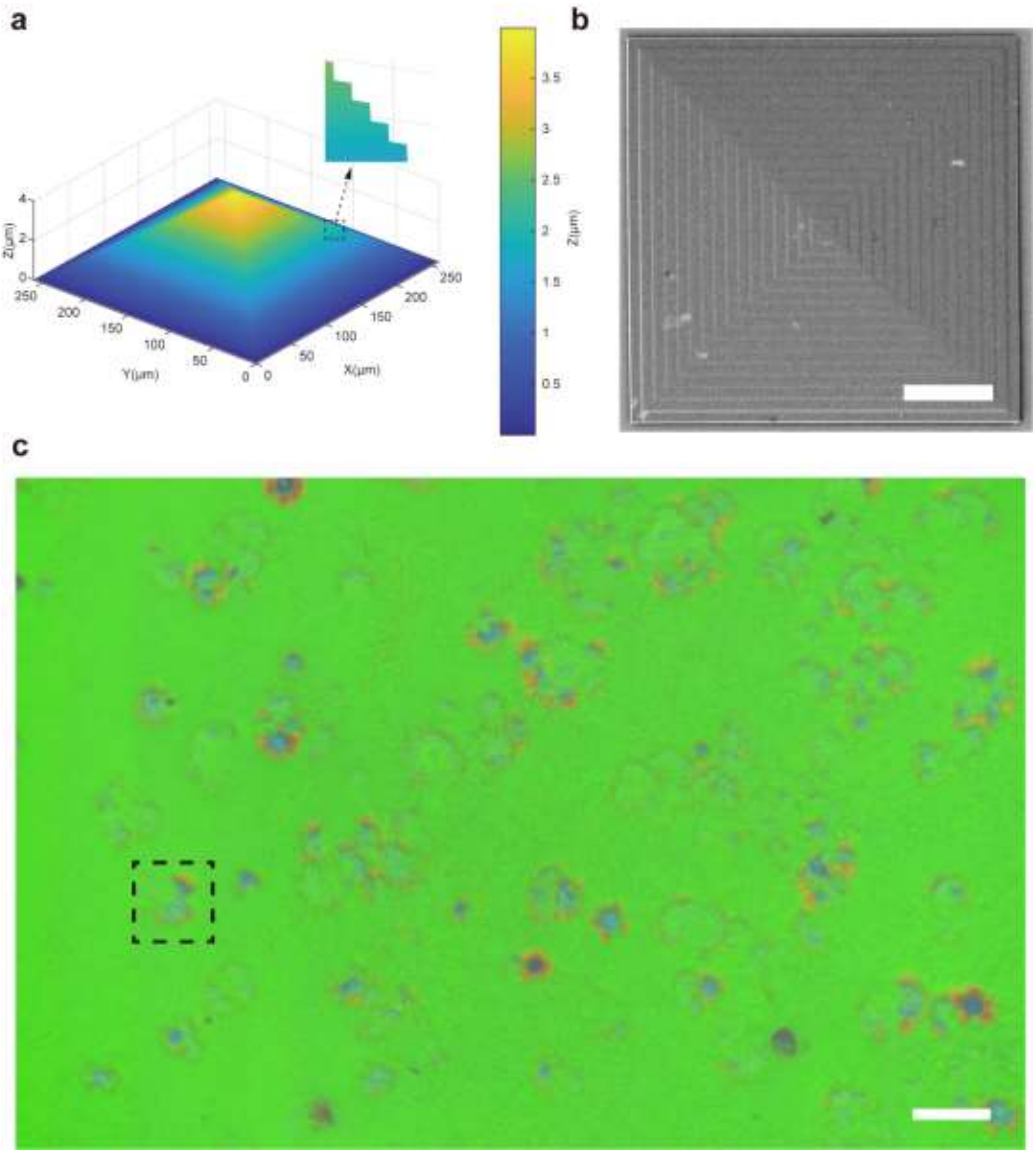

Supplementary Figure 3 (a) Schematic diagram of the 3D printed pyramid structure. **(b)** SEM image of the 3D printed pyramid sample. Scale bar, 50 μm. **(c)** PCCFM images of dozens of MDCK cells grown on PCS. Scale bar, 100 μm.

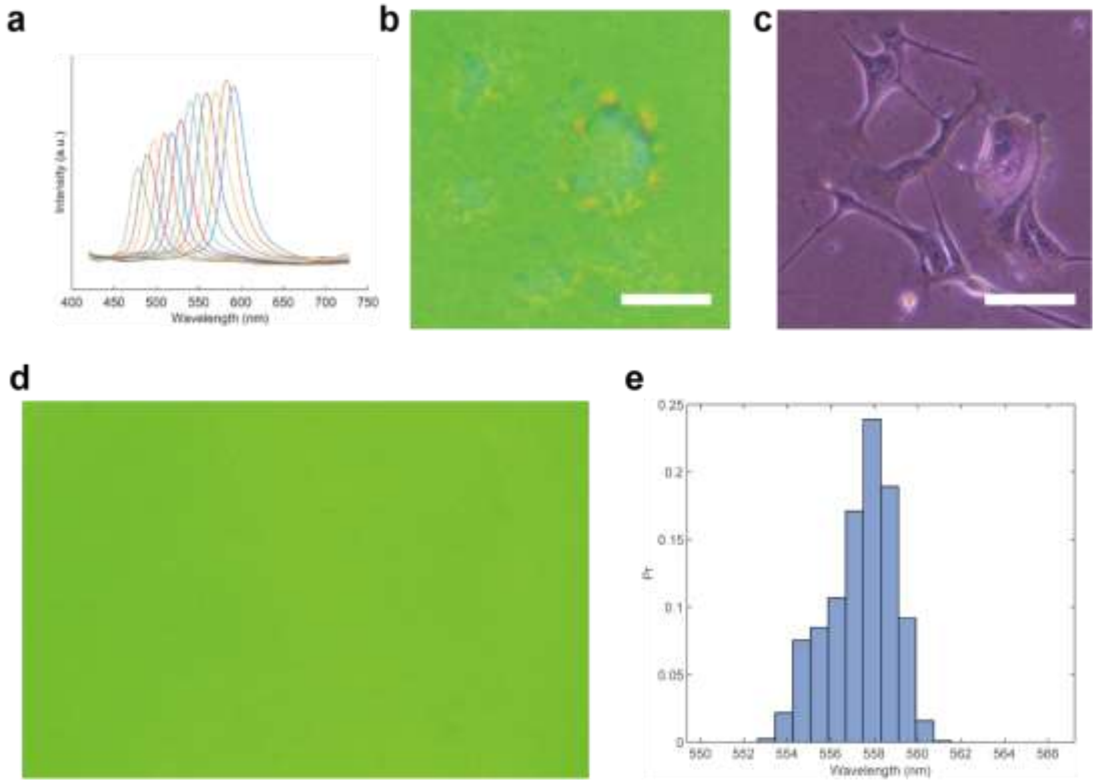

Supplementary Figure 4 Establishment of the relationship between hue and PCS reflection peak position. **(a)** The reflection spectrum along the dashed line in Fig. 2b and 2c was obtained using hyperspectral scanning. The PCCFM image **(b)** and the bright field image **(c)** of the MDCK cells. Scale bar, 25 μm. A photograph of a typical 10x magnification full-field view of PCCFM **(d)** and its corresponding distribution map of reflection peak wavelengths **(e)** obtained through pixel-wise analysis based on hue-wavelength relationship, with a standard deviation of strain of 0.0027.

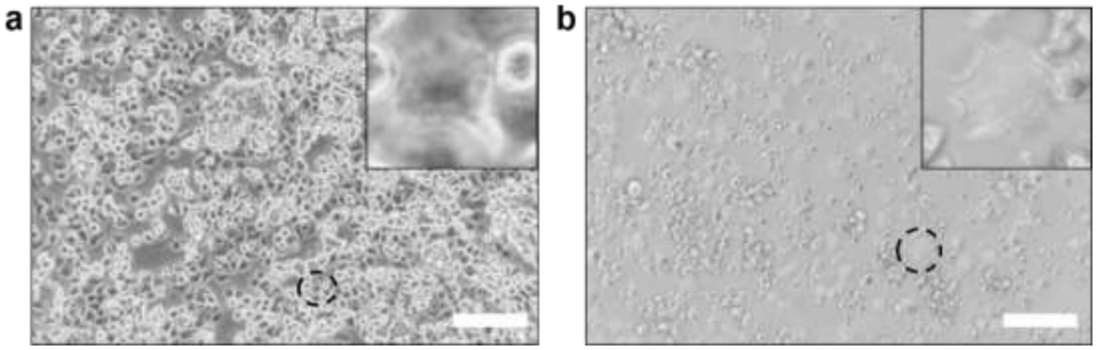

Supplementary Figure 5 The bright field images of the cells grown on PCS. NCI-H460 cells **(a)** and primary neonatal rat cardiomyocytes **(b)** grown on the PCS, scale bar 200 μm.

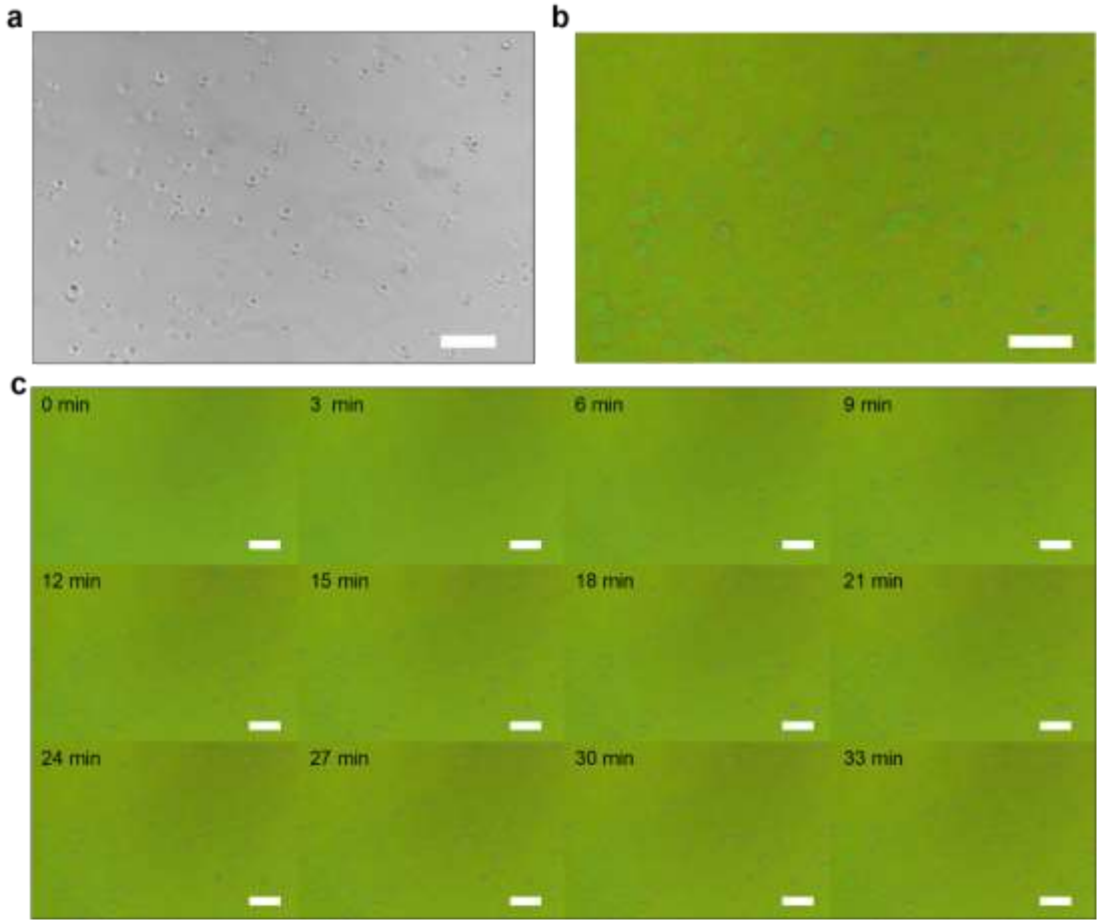

Supplementary Figure 6 Image sequence of the cell attachment process. All photos were taken with the microscope's 10 × objective lens. The phase contrast image at minute 33 is shown in (**a**), and the corresponding PCCFM image is shown in (**b**). Scale bar, 100 µm. The whole PCCFM image sequences are shown in (**c**). Scale bar, 100 µm.

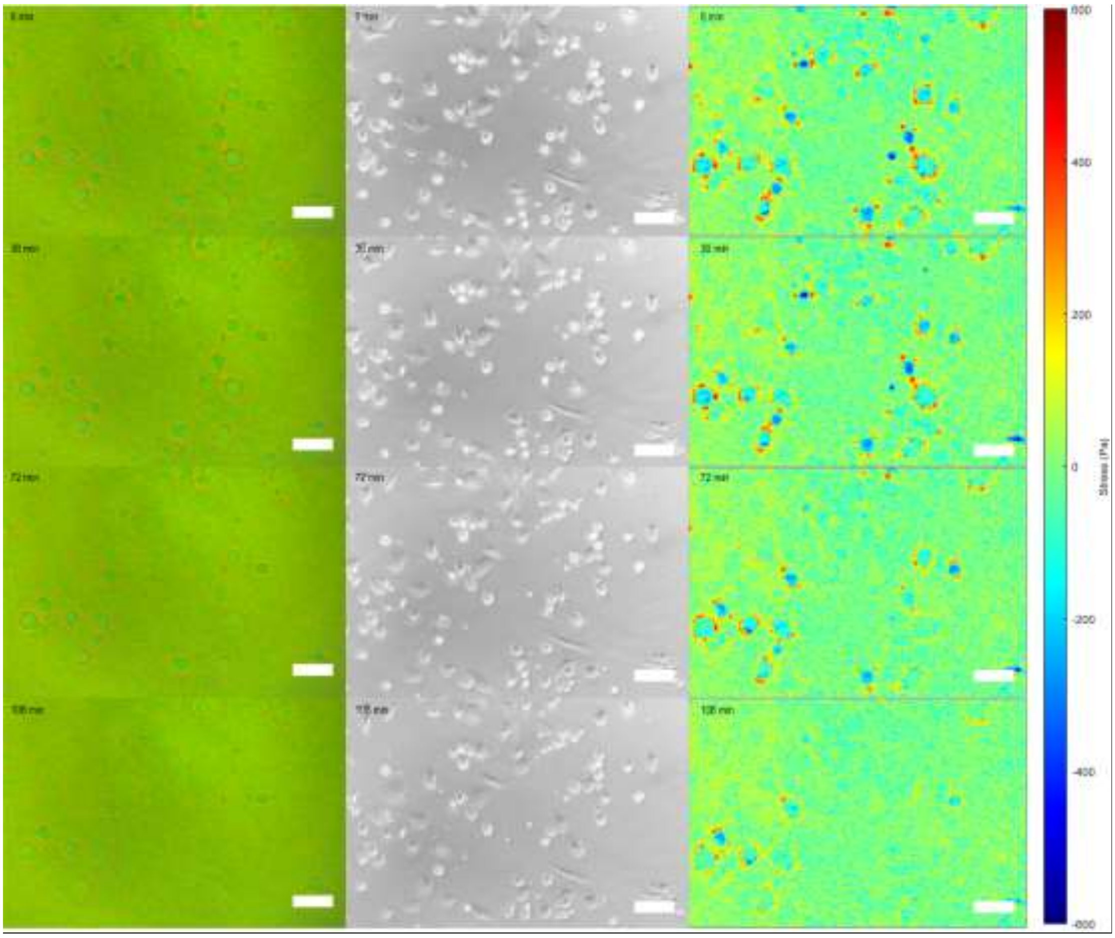

Supplementary Figure 7 PCCFM, bright field image sequences, and corresponding stress maps of the MDA-MB-231 cells treated with paclitaxel. Scale bar, 50 μm.

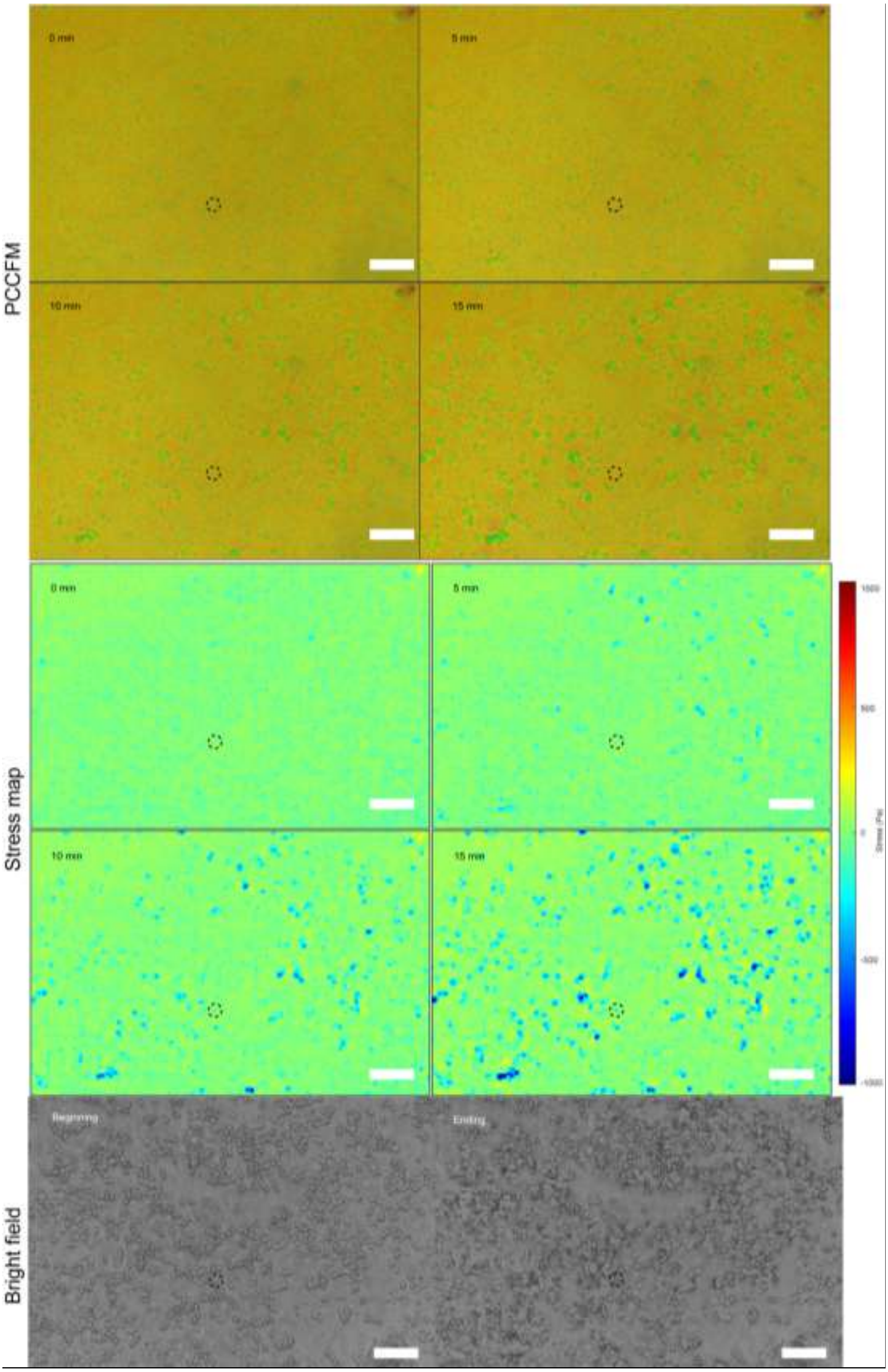

Supplementary Figure 8 PCCFM image sequences and corresponding stress maps of the

HEK293V cells treated with nocodazole. The bright-field image of the beginning moment and the ending of the experiment are also presented. Scale bar, 100 μm.

To demonstrate the correlation between TFM and PCCFM measurement results, 200 nm fluorescent nano-beads were incorporated into the photonic crystal hydrogel to fabricate a substrate that enabled simultaneous TFM and PCCFM measurements. After seeding MDCK cells on green PCS for 4 hours, PCCFM images and phase-contrast images were acquired, followed by PCCFM analysis. Subsequently, while acquiring fluorescent images, the cells were detached from the substrate and TFM analysis was conducted, including particle tracking using Matlab's PIVLab app and force field calculation utilizing ImageJ's FTTC plugin[1-5]. Consistent with previous observations, the PCCFM images showed a red shift at the outer edge of the cell periphery due to the upward pulling traction forces of focal adhesions, while a blue shift was observed at the inner side of the cells due to downward pushing. Comparing the maximum pulling forces measured by PCCFM with those by TFM, the peak value by PCCFM was approximately half of the TFM measurement. The normalized vertical stress by PCCFM and the traction force by TFM had a structural similarity index (SSIM) of 0.62 for the regions outlined by the white dashed lines in Supplementary Figure 9(e) and (f).

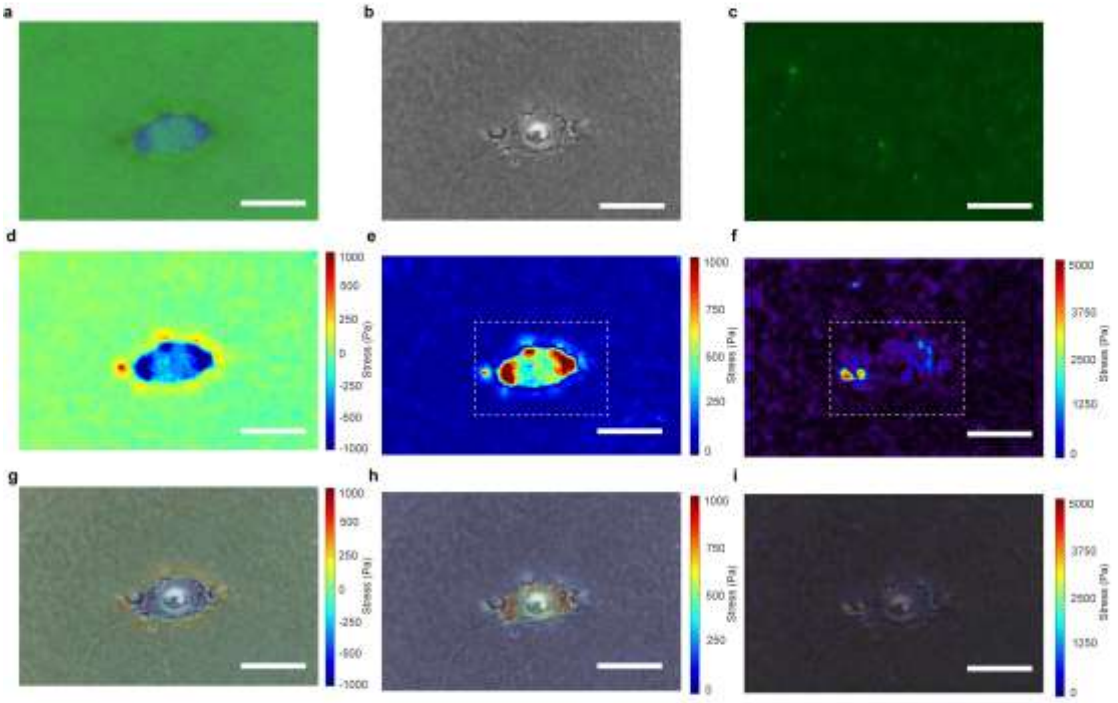

Supplementary Figure 9 Comparison of TFM and PCCFM measurement results. **(a)** PCCFM image of the cell. **(b)** Corresponding bright-field image. **(c)** Fluorescent nano-beads in the substrate. **(d)** Vertical stress analysis of (a). **(e)** The absolute value of (d). **(f)** TFM stress field. **(g)** Overlay of (a) and (b). **(h)** Overlay of (b) and (e). **(i)** overlay of (f) and (b). Scale bar, 100 μm.

References

1 Thielicke, W. & Sonntag, R. Particle Image Velocimetry for MATLAB: Accuracy and enhanced algorithms in PIVlab. *Journal of Open Research Software* **9** (2021).

2 Stamhuis, E. & Thielicke, W. PIVlab–towards user-friendly, affordable and accurate digital particle image velocimetry in MATLAB. *Journal of open research software* **2**, 30 (2014).

3 Thielicke, W. The flapping flight of birds. *Diss. University of Groningen* (2014).

4 Garcia, D. A fast all-in-one method for automated post-processing of PIV data. *Experiments in fluids* **50**, 1247-1259 (2011).

5 Martiel, J.-L. *et al.* in *Methods in cell biology* Vol. 125 269-287 (Elsevier, 2015).
