# Supplementary material for: Imaging cellular forces with photonic crystals
Source: Nat Commun. 2023 Nov 14;14:7369. doi: 10.1038/s41467-023-43090-9 (PMC10646022; doi:10.1038/s41467-023-43090-9)
Supplement: Supplementary file 3 — Description of Additional Supplementary Files [file 41467_2023_43090_MOESM3_ESM.pdf]

Title: Supplementary Movie 1

Description: Video generated from PCCFM image sequences recording the attachment and spreading process of MDA-MB-231 cells.

Title: Supplementary Movie 2

Description: The original video of the myocardial sheet in PCCFM mode cultured for 7 days.

Title: Supplementary Movie 3

Description: The original video of the myocardial sheet in PCCFM mode treated by drugs.
